# Focus on pelvic trauma

**DOI:** 10.1007/s00068-021-01823-9

**Published:** 2021-11-29

**Authors:** Pol Maria Rommens, Daniel Wagner

**Affiliations:** Department of Orthopaedics and Traumatology, University Medical Center of Johannes Gutenberg-University Mainz, Mainz, Germany

High-energy pelvic injuries remain a reason for intense scientific debates among physicians involved in trauma care. The primary goal is to save lives in severely injured patients and performing focused successful damage control surgery. After and aside of treating patients in the critical phase, further stabilization and especially their patient reported outcomes are of increasing interest. Continuous points of dispute are the best sequence of diagnostic and therapeutic measures and the usefulness of minimal-invasive surgical techniques for definitive stabilization.

In this focus on issue, six original articles highlight different new aspects of diagnosis and treatment. They cover aspects of early management and definitive treatment. Kim et al. from Korea [1] compared the survival rates after 28 days of 260 patients, who have been treated with or without trans-arterial embolization (TAE) for a pelvic injury. Survival rate of patients, who have been treated with TAE was 93.9%, whereas patients without TAE had a survival rate of only 86.2%. The difference was significant. This data clearly shows that TAE—among other procedures and with the right indication—has an important, life-saving role in the resuscitation phase of pelvic trauma patients.

External fixation is a proven method of provisional and definitive stabilization of the pelvic ring. Pins can be inserted at the iliac wing or in the supra-acetabular area. In case of optimal pin insertion, adequate stability is obtained in the anterior pelvis. The importance of correct pin placement is also given by the increasing use of minimal-invasive fixation techniques in geriatric pelvic fractures [2, 3]. New insights in the anatomical characteristics of the ilium give us precise recommendations for correct pin insertion. Von Glinski et al. [4] calculated on 9 anatomical specimen the relation between the thin monocortical area in the middle of the iliac wing and different bone trajectories in the ilium body. They could prove that a deviation of less than 5° in the supra-acetabular trajectory may already perforate the monocortical area, leading to less stable anchorage of the pins, iatrogenic fractures or damage to the nearby supragluteal vascular bundle. These findings do support a thorough preoperative planning and careful operation technique for external or internal fixation of the pelvic ring. Shan et al. [5] performed another study on the same subject. CT-data of 100 uninjured pelvis were converted into 3D-models and all supra-acetabular corridors from the anterior inferior (AIIS) to the posterior superior iliac spines (PSIS) were precisely calculated. The optimal insertion point at the AIIS is at its outer lower part, the ideal direction of the screws about 30° towards medially and cranially. In their finite element analysis, stability after insertion of a semi-length screw was similar to the full-length screw.

In the following original papers, the authors investigate the possibilities of conventional and new technologies for preoperative planning and intra-operative control of reduction and implant positioning. Rommens et al. [6] looked at the safety of 2D-fluoroscopy based iliosacral screw osteosynthesis in patients below 65 years of age. 207 iliosacral screws were inserted in 101 procedures. A minimal-invasive procedure was performed in 77.5% of the patients. 12 early and 5 late operative revisions were necessary. Screw penetration was detected in postoperative CT in 20 cases, all of them in double screw osteosynthesis of S1. The authors conclude that 2D-fluoroscopy based iliosacral screw osteosynthesis remains a safe method in times of computer navigation, provided that a thorough preoperative analysis of the anatomy of the upper sacrum and planning of the screw localization is done.

Wang et al. [7] assessed the influence of 3D printing for open reduction and internal fixation of pelvic fractures through a meta-analysis of randomized controlled trials and prospective comparative studies. Five studies were collected with 174 patients in the 3D printing group and 174 in the conventional group. The authors found significant differences of operation time, intra-operative blood loss and postoperative complications. Quality of pelvic fracture reduction and functional outcome were superior as well. The authors conclude that 3D printing technology is an important support in understanding the particular features of each pelvic fracture and for preparing the operative procedure.

In this focus on pelvic trauma, different modalities for emergency treatment, provisional stabilization and definitive care are discussed. Pelvic trauma remains a challenging entity, for which resuscitation, preoperative planning for definitive surgery, limitation of radiation and methods of minimal-invasive surgery are important aspects. The original papers of this focus on may guide the individual trauma surgeons in their choice on how to proceed in their specific hospital setting. We wish you interesting reading and new insights thereafter.

Prof. Dr. Dr. h. c. Pol M. Rommens

Guest Editor

PD Dr. Daniel Wagner

Co-Guest Editor
